# A bibliometric analysis of research trends for missing persons globally and in East Asia from 2000 to 2021

**DOI:** 10.1093/fsr/owad041

**Published:** 2024-02-25

**Authors:** Zixuan Zeng, Ishmael Dikeledi, Yehui Lv

**Affiliations:** School of Basic Medical Sciences, Shanghai University of Medicine & Health Sciences, Shanghai, China; Institute of Wound Prevention and Treatment, Shanghai University of Medicine & Health Sciences, Shanghai, China; School of Basic Medical Sciences, Shanghai University of Medicine & Health Sciences, Shanghai, China; School of Basic Medical Sciences, Shanghai University of Medicine & Health Sciences, Shanghai, China; Institute of Wound Prevention and Treatment, Shanghai University of Medicine & Health Sciences, Shanghai, China

**Keywords:** bibliometrics, missing persons, forensic sciences, forensic humanitarian, forensic anthropology, global trend, East Asia, English literature

## Abstract

A bibliometric analysis was performed to explore the current research status and development trends for missing persons globally and in East Asia and to identify research hotspots and frontiers relating to this topic. A search was conducted to identify relevant literature on missing persons using the Web of Science Core Collection database for the period 2000–2021. Subsequently, a knowledge map was constructed using CiteSpace software to perform a visual analysis of the distribution of authors and institutions, journals, and national/regional distribution; citation frequency; high-frequency keywords; and emerging research hotspots. The results showed firstly that discussions on missing persons and related topics in East Asia are held at the regional scale. There is a paucity of research on this topic, which has been conducted on a limited scale, lacks depth and possibly innovation, and entails limited discussion in this region. Secondly, there is a lack of social science research on missing persons and related topics worldwide. Thirdly, relevant research in East Asia should continue to preserve its own characteristics, effectively addressing current issues and enabling more people to participate in social science-oriented discussions focusing on the topic of missing persons. This approach provides a promising direction for pursuing the sustainable development of the topic of missing persons.

**Key points:**

- The strengths and weaknesses of current research on missing persons in East Asia were identified by comparing the respective literatures on missing persons and related topics in East Asia and worldwide during the period 2000–2021.
- Bibliometric analysis was performed using CiteSpace visual charts to explore keywords, authorship and co-authorship, intercountry collaboration, and other relevant co-citationities.
- Insights were obtained on current research breakthroughs relating to the topic of missing persons, and cutting-edge social science research on this topic was identified.

## Introduction

Issues relating to human remains and missing persons have received considerable attention within the global forensic anthropology literature along with technological innovations and widely discussed ethical issues relating to these topics. However, a preliminary bibliometric survey and analysis of the relevant literature published over a period of more than 20 years revealed that in East Asia (i.e. China, Japan, Korea, and Mongolia), discussions on this and related topics are limited. Natural disasters such as typhoons with strong winds, very heavy rainfall and storm surges as well as flooding (continuous heavy rainfall or short periods of heavy rainfall), droughts, cold waves, volcanic eruptions, and earthquakes occur relatively frequently in East Asia [1]. Moreover, there is a historical legacy of wars in this region. Consequently, the phenomenon of missing and unidentified persons in this region is not uncommon. Notwithstanding these issues, a gap remains in the research on this topic in East Asia.

A certain amount of cutting-edge research has been conducted on the identification of missing persons worldwide. Examples include the use of digital fingerprinting in disaster victim identification (DVI) settings [2] and massively parallel sequencing (MPS), which is used for highly variable regions of human mitochondrial DNA, identifying DNA from damaged biological samples [3], and for examining exhumed human remains, especially in areas where there is no evidence of human remains. Ground-penetrating radar [4] has also been used, especially in undocumented areas where human remains are buried. However, the findings of this study revealed a paucity of social science research on this topic within the literature despite its increasing importance within the social sciences, as evidenced by recent discussions in the social sciences [5–8]. Furthermore, very few analyses, evaluations, and summaries of research findings on issues relating to human remains and missing persons exist within the literature.

Bibliometric analysis is a widely used quantitative method for estimating relevant, previously conducted research activities, enabling the content of the literature to be analysed and new trends in a given field to be predicted. Bibliometrics has contributed to research trends in forensic science and legal medicine [9, 10], such as forensic anthropology [11], forensic entomology [12], forensic genetics [13], and medical malpractice [10]. However, in the field of missing person or forensic humanitarian, to the best of our knowledge, this bibliometric analysis is the first attempt. The recommendations of this study are to conduct a systematic assessment of the research content, trends, and topical issues relating to human remains and missing persons from 2000 to 2021, with the aim of capturing patterns of collaboration between countries/regions, institutions, and authors and exploring future research directions in this field. Such an assessment would enable differences between East Asia and the world as a whole relating to the issue of human remains and missing persons to be identified and would provide a reference for continued research on this topic in East Asia.

## Materials and methods

### Search strategy and data collection

For this retrospective study, data were obtained on 1 July 2022 from the Web of Science Core Collection (WOSCC) using the Science Citation Index Expanded and the following search formula:

(TS = (missing person) OR TS = (missing population) OR TI = (missing) OR TS = (missing people) OR TS = (disaster missing) OR TS = (missing disorder) OR TS = (missing conflict) OR TS = (migrant missing) OR TS = (disappearing population) OR TI = (disappearing person) OR TI = (vanishing population) OR TI = (surviving population) OR TS = (survivor) OR TS = (disaster victim identification) OR TS = (DVI) OR TS = (identification of victim) OR TS = (unidentified human) OR TS = (investigation on missing person) OR TS = (human remains) AND ( TI = (forensic or legal medicine) OR TS = (forensic^*^ or legal medicine) OR AB = (forensic or legal medicine) OR AK = (forensic or legal medicine) OR KP = (forensic? or legal medicine)).

The terms were refined by excluding document types: (editorial material OR letter OR data paper OR book chapter OR proceedings paper OR retraction OR meeting abstract OR correction OR news item OR retracted publication), and languages (English).

The range of publication dates for articles included in the search was 1 January 2000 to 31 December 2021.

### Data analysis

A total of 3 381 articles were searched worldwide, of which 238 focused on the East Asian region. The search results were downloaded in full in TXT format and subsequently exported to a Microsoft Excel 2019 (Redmond, USA) spreadsheet to analyse general statistics and to conduct a further analysis using CiteSpace 5.8.R3 (https://citespace.podia.com/). The data were aggregated as secondary data in a public database.

An analysis of the numbers and types of papers, years of publication, journals, authors, institutions, countries and regions, funding bodies, numbers of citations, and the *h*-index was carried out using WOSCC’s results analysis tool. Temporal trends concerning the annual growth rate and relative growth rate over a certain interval within the literature were calculated using a Microsoft Excel 2019 spreadsheet. CiteSpace, which is a specialist software used for analysing and visualizing bibliometric networks, was applied in this study for the visual bibliometric analysis, which produced a map of keyword clusters, a timeline view of keywords, and a burst keywords map for relevant literature worldwide, with countries/regions, institutions, and authors. The above graphs were also produced for the East Asian region for comparison purposes. Accordingly, the CiteSpace parameters were as follows. For time slicing, the cutoff time point for the analysis, which covered the period 2000–2021, was 1 year. For selection criteria, the g-index was selected, and the scale factor was set at *k* = 25 to highlight important literature. For the node types, authors, institutions, countries (regions), and keywords were selected for the visual analysis and generation of co-occurrence plots. The system’s default settings were used for the remaining parameters.

## Results and discussion

### Number of documents and publication trends

**Figure 1:**
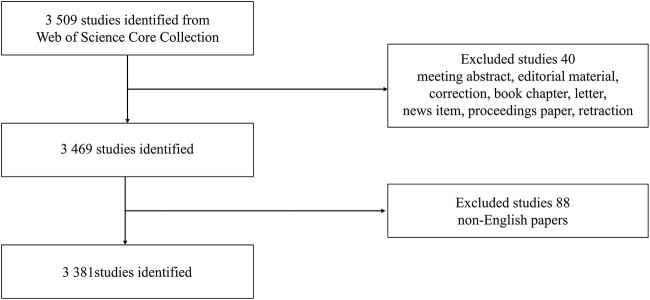
Screening process for studies included in this bibliometric research.

Initially, 3 509 eligible documents were obtained and screened using the process shown in Figure 1. Of these publications, 88 were written in languages other than English: 39 were in German (44.3%); 12 were in Spanish (13.6%); 11 were in French (12.5%); 4 each were in Russian (4.5%) and in unspecified languages (4.5%); 3 each were in Chinese (3.4%), Polish (3.4%), and Serbian (3.4%); 2 each were in Hungarian (2.2%), Portuguese (2.2%), and Turkish (2.2%); and 1 each were in Italian (1.1%), Korean (1.1%), and Slovene (1.1%) (Number are rounded so the proteges may not add to 100%). What is more, 40 were meeting abstract, editorial material, correction, book chapter, letter, news item, proceedings paper, or retraction. Ultimately, 3 381 eligible documents were obtained, of which 238 were from the East Asian region.

**Figure 2:**
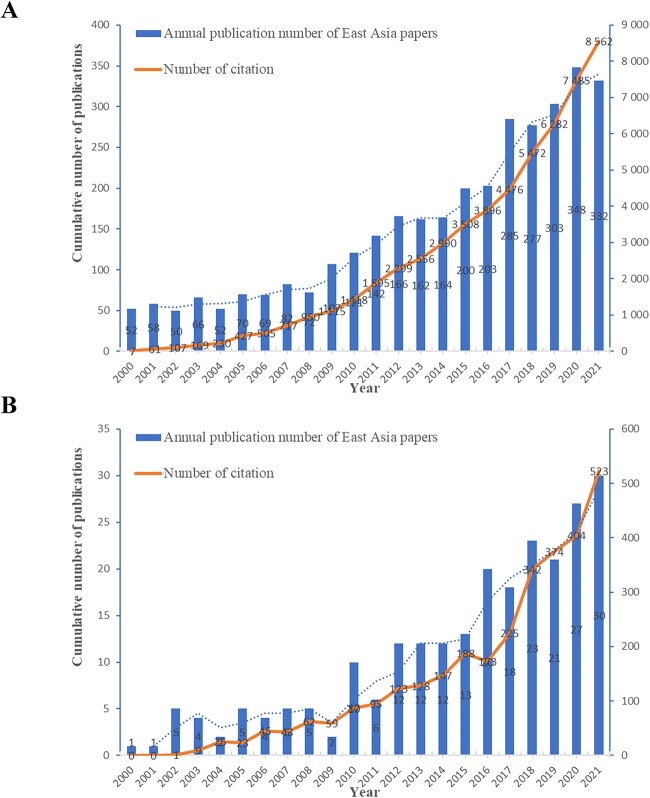
Trends in publication of missing persons worldwide (A) and in East Asia (B).

The global and East Asian literature were aggregated, and publication trends were plotted, as shown in Figure 2. It can be seen that during the period 2000–2021, the literature on this topic showed a steady increasing trend both globally and in East Asia; it is similar to the results of some bibliometric studies in forensic science [10, 13]. What is more, the increasing trend is more apparent in East Asia; this may reflect the significant development of research interest and scientific input in East Asian countries over the past two decades. These findings may indicate that there is still potential, but we should also recognize that compared with the world, there are still many shortcomings.

Citations in the literature in Figure 2A will be discussed later in the article.

### Literature-producing countries/regions and literature-producing institutions

**Figure 3:**
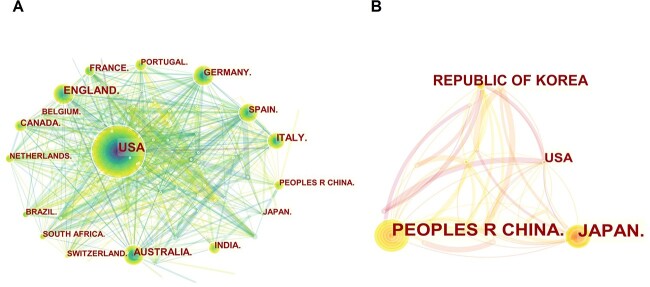
Network maps of national and regional sites of literature production worldwide (A) and in East Asia (B).

**Figure 4:**
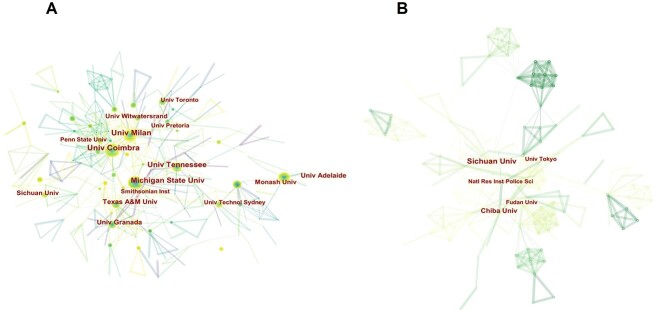
Network maps of institutional sites of literature production worldwide (A) and in East Asia (B).

CiteSpace was used to generate network maps of national/regional and institutional literature, as shown in Figures 3 and 4 and Table 1. It can be seen that on a global scale, the USA produced the largest body of research literature (29.8%). Within Europe, the UK produced the most literature (10.6%), followed by Italy (7.2%), Germany (6.1%), Spain (4.9%), France (3.5%), and Portugal (3.1%). Within Oceania, Australia (8.6%) produced the most literature after the USA and the UK. Within Asia, India (4.2%) was ranked in the eighth position in terms of its literature output. Thus, Australia and India were the only non-European and non-American countries to feature in the list of the top 10 countries in terms of their literature outputs. Of the East Asian countries, Japan (3.0%) had the most relevant literature, followed by China (2.9%), with these two countries together accounting for 84.0% of the literature published on human remains and missing persons within East Asia. The characteristics of research in East Asia were also influenced by the size of China’s population, which is the largest population globally (according to countrymeters, 2022-01-01) and by Japan’s vulnerable status as one of the most natural disaster-prone countries in the world [1]. Figure 4 shows that the institutions currently publishing literature on the topic under investigation are mainly located in Europe and the USA, with these institutions connected by complex common threads, indicating close collaborations among mainstream institutions. The most prominent East Asian institutions including Institute of Forensic Science (now renamed as Academy of Forensic Science), Ministry of Justice, PRC, Sichuan University and Josai University. However, only a few collaborative common threads were evident, as this institution only works with a limited number of other institutions, almost all of which are Chinese institutions. This example is illustrative of the collaborative activities of most East Asian institutions as well as the nature of their collaboration, which is localized and with single institutions.

**Table 1:** Ranking of countries and institutions that published articles worldwide and in East Asia (Top 10)

Rank	Worldwide (3 381 papers)				East Asia (238 papers)			
	Country/Region		Institutions		Country/Region		Institutions	
	Name	Count	Name	Count	Name	Count	Name	Count
1	USA	1007	Universidade de Coimbra	75	Japan	102	Institute of Forensic Science, Ministry of Justice, PRC^*^	27
2	UK	359	Udice French Research Universities	68	China	98	Sichuan University	25
3	Australia	290	University of London	65	Republic of Korea	31	Josai University	17
4	Italy	242	University of Milan	58	USA	22	University of Tokyo	13
5	Germany	206	University of Tennessee System	58	Germany	13	Natl Res Inst Police Sci	12
6	Spain	167	State University System of Florida	55	UK	10	Fudan University	11
7	Canada	163	Michigan State University	53	Taiwan, China	10	Seoul National University	10
8	India	142	University of California System	51	Canada	4	National Forensic Service	9
9	France	120	University of Tennessee Knoxville	51	France	4	Osaka Metropolitan University	9
10	Portugal	104	University of Adelaide	49	Netherlands	4	Central South University	8

^*^Now renamed Academy of Forensic Science.

### Journals

Tables 2 and 3 present statistics on the top 10 journals published globally and in East Asia, respectively. It shows that the following journals featured among the top 10 publications both at the global level and in East Asia: *Forensic Science International*, *Journal of Forensic Sciences*, *International Journal of Legal Medicine*, *Forensic Science International: Genetics*, *Journal of Forensic and Legal Medicine*, and *Legal Medicine.* Other journals included among the top 10 journals at the global level were *Science & Justice, American Journal of Forensic Medicine and Pathology, Forensic Science Medicine and Pathology and Australian Journal of Forensic Sciences*. *Forensic Science International* and *Forensic Science International: Genetics* occupied the same positions in both tables for the global and East Asian publications (1 and 4, respectively). *American Journal of Forensic Medicine and Pathology* and *Australian Journal of Forensic Sciences* were probably in the top 10 list because of the high number of studies on missing persons in the USA and Australia (first in the USA and third in Australia), which is one of the reasons for the high rank of the two magazines. The *h*-index values for *Romanian Journal of Legal Medicine* and *Forensic Science Research*, which are among the top 10 journals in East Asia in terms of number of publications, were 44 and 23, respectively. These values for the two journals were ranked 13 and 14, respectively, among the 14 journals listed in the two tables, indicating a possible lack of innovativeness of some of the relevant literature in East Asia.

**Table 2:** Top 10 journals promoting scientific research on missing persons globally (Total *N* = 3 381)

Rank	Journal	Count	Percentage of total (%)	*h*-index	Self-citation rate (%)
1	*Forensic Science International*	490	14.5	120	17.0
2	*Journal of Forensic Sciences*	472	14.0	98	11.2
3	*International Journal of Legal Medicine*	200	5.9	83	20.0
4	*Forensic Science International: Genetics*	142	4.2	75	48.0
5	*Journal of Forensic and Legal Medicine*	117	3.5	47	13.4
6	*Science & Justice*	63	1.9	42	21.0
7	*American Journal of Forensic Medicine and Pathology*	53	1.5	57	9.8
8	*Forensic Science Medicine and Pathology*	52	1.5	37	14.1
9	*Legal Medicine*	49	1.4	44	10.9
10	*Australian Journal of Forensic Sciences*	48	1.4	23	10.2

**Table 3:** Top 10 journals promoting scientific research on missing persons in East Asia (Total *N* = 238)

Rank	Journal	Count	Percentage of total (%)	*h*-index	Self-citation rate (%)
1	*Forensic Science International*	28	11.8	120	17.0
2	*International Journal of Legal Medicine*	26	10.9	83	20.0
3	*Journal of Forensic Sciences*	21	8.9	98	11.2
4	*Forensic Science International: Genetics*	20	8.4	75	48.0
5	*Scientific Reports*	11	4.7	213	4.3
6	*Legal Medicine*	9	3.8	44	10.9
7	*Electrophoresis*	4	1.7	57	17.9
8	*Journal of Forensic and Legal Medicine*	4	1.7	47	13.4
9	*Romanian Journal of Legal Medicine*	4	1.7	44	13.5
10	*Forensic Science Research*	3	1.3	23	7.7

**Figure 5:**
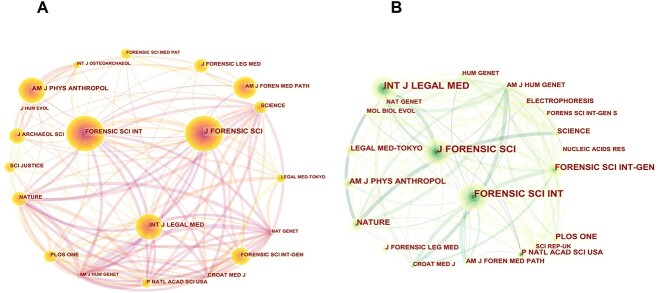
Co-citation journal maps worldwide (A) and in East Asia (B).

Co-citation journal maps were produced using CiteSpace, as shown in Figure 5. There was a high degree of similarity in the cited literature between East Asian and world literature. This result may also reflect the fact that current discussions on this topic primarily feature in three journals: *Journal of Forensic Sciences*, *Forensic Science International*, and *International Journal of Legal Medicine*.

### Collaborations and funds

**Figure 6:**
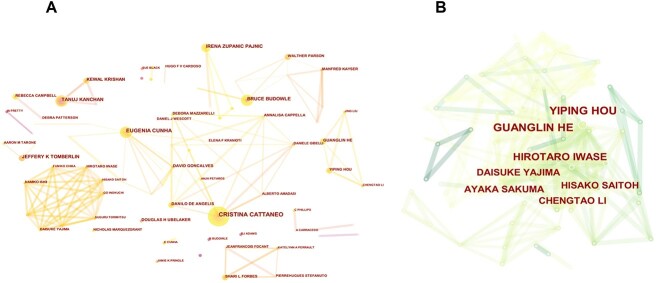
Network map of active authors contributing to missing persons worldwide (A) and in East Asia (B).

Co-citation maps of collaborations among authors were also produced using CiteSpace, as shown in Figure 6. Evidently, there are deeper collaborations between authors worldwide, with collaboration co-citationities in East Asia being much thinner in comparison. This difference may be attributable to the distinct cultural characteristics of the East Asian region, which also account for linguistic and other differences between authors from countries (regions) in East Asia and other countries (regions). Furthermore, geographical and climatic differences should also be considered.

Statistics on the relevant global and East Asian funding bodies are shown in Tables 4 and 5. The tables show that two of the top 10 funding agencies, namely, the National Natural Science Foundation of China (1.6%) and the Ministry of Education, Culture Sports Science and Technology in Japan (0.9%), are from the East Asian region. This finding reveals a certain level of interest in the topic of missing persons in East Asia and some willingness to fund efforts to address this issue. Within East Asia, five of the top 10 grant-making agencies are located in China and three are located in Japan. This statistic shows that Japan and China provide the most support for research on this topic within East Asia, while producing the most relevant literature in this region.

**Table 4:** Top 10 funding agencies globally (Total funded paper: *N* = 3 381)

Rank	Funding agency	Founded paper: *n* (%)
1	European Commission	99 (2.9)
2	US Department of Health Human Services	56 (1.7)
3	US National Institutes of Health	55 (1.6)
4	National Natural Science Foundation of China	54 (1.6)
5	Portuhguese Foundation for Science and Technology	48 (1.4)
6	US National Science Foundation	35 (1.0)
6	US National Institute of Justice	34 (1.0)
8	Spanish Government	31 (0.9)
9	Ministry of Education Culture Sports Science and Technology Japan	30 (0.9)
10	Australian Research Council	28 (0.8)

**Table 5:** Top 10 funding agencies in East Asia (Total funded paper: *N* = 238)

Rank	Funding agency	Founded paper: *n* (%)
1	National Natural Science Foundation of China	54 (22.7)
2	Ministry of Education Culture Sports Science and Technology, Japan	29 (12.2)
3	Japan Society for The Promotion of Science	24 (10.1)
4	Grants-in-Aid for Scientific Research, Japan	14 (5.9)
5	Fundamental Research Funds for The Central Universities, China	10 (4.2)
6	National Research Foundation of Korea	8 (3.4)
6	National Key Research and Development Program of China	6 (2.5)
8	Science Technology Commission of Shanghai Municipality	6 (2.5)
9	Ministry of Education, Science Technology, Republic of Korea	5 (2.1)
10	National High Technology Research and Development Program of China	4 (1.7)

### Keywords in the literature

**Figure 7:**
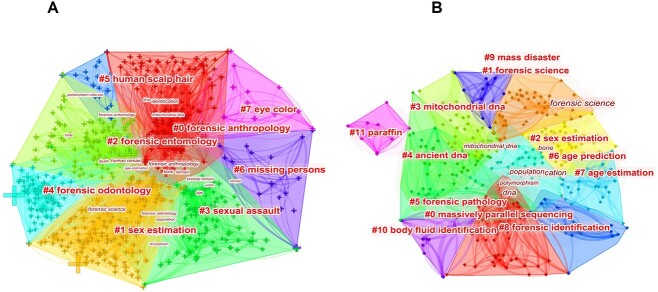
Maps of keyword clusters worldwide (A) and in East Asia (B).

CiteSpace was used to create keyword cluster maps for the global and East Asian literatures, respectively, as depicted in Figure 7. The figure shows that mainstream voices are currently focused on issues relating to the identification of human remains. For example, high-tech means are used to identify DNA and RNA of human remains and hence to obtain identity-related information at the global and East Asian scales. Studies have also been conducted to identify skeletal remains from various types of bones [14–16], focusing on the role of bones, such as skulls and teeth, as important identifiers. This information is used along with the identification of personal information such as the sex [17], age [18], and race [19] of the deceased, which further enhances the accuracy of the identification of the remains. The keywords in the East Asian literature reveal a high degree of similarity to those used worldwide, but because of the special circumstances of the East Asian region [1], the categories of mass catastrophe and MPS were included in the clustering of keywords in the East Asian literature [20–25], which is one of the distinct features of the East Asian research on human remains and missing persons. However, it is clear from these charts that a social science perspective is missing in the research on human remains and missing persons both worldwide and in East Asia, with only one relevant cluster, namely, “sexual assault” featuring among the keywords.

**Figure 8:**
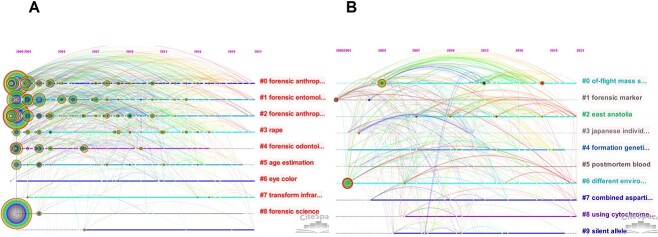
Timeline view of keywords analysis worldwide (A) and in East Asia (B).

CiteSpace was used to create a timeline view of the keywords in the global and East Asian literature, as shown in Figure 8. The timeline view provided a sketch of the relationships between clusters and the historical span of the literature in a given cluster [26]. The denser concentration of hot terms in the global map of keyword timeline clustering attests to the depth and breadth of research on the topic of human remains and missing persons worldwide. At the global scale, throughout the period 2000–2021, topics from the disciplines of forensic anthropology and forensic entomology were hot research topics in this field, entailing explorations of various topics relating to the identification of human remains. The “rape” cluster also appears in the map, represented by several studies in the humanities and social sciences that have explored the impacts of sexual assault, on female victims [27–29], rapists’ and victims’ psychological profiles [30], and adolescent victims and perpetrators [31, 32]. In addition, a number of studies have focused on the identification of rape victims’ remains [33]. Comparing the timeline views of keywords in the global and East Asian literature, it is apparent that East Asian research reached a certain scale significantly later than research at the global level. Moreover, the scale of research in East Asia has been relatively limited, with studies focusing more on MPS [23–25] and East Asian ethnic identification [24, 34, 35].

**Figure 9:**
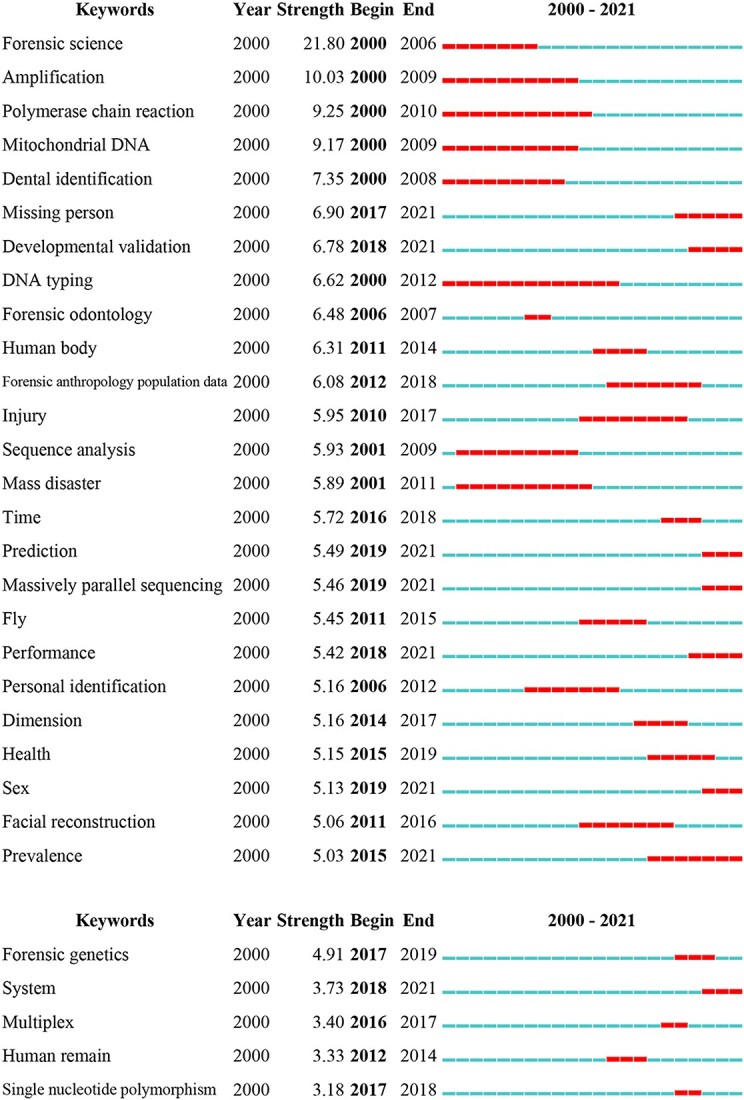
Burst keywords worldwide (A) and in East Asia (B).

Figure 9, which depicts burst keywords in the global and East Asian literature, was produced using CiteSpace (“Strength” indicates the degree of prominence, with higher levels representing more prominent discussion at a given time.). Burst keywords can aid in an analysis of the evolution of research hotspots [26]. For the same burst term model values, there were 25 burst terms worldwide and 5 burst terms in East Asia during the period 2000–2021. The number of emergent words in East Asia was much lower than the global number, reflecting the limited exploration of this topic in East Asia. In addition, the durations of discussions featuring topical words were shorter in East Asia, lasting, on average, 2.8 years (a median value of 3 years) compared with 7.8 years (a median value of 6 years) at the global scale, and the years of emergence of topical words also lagged behind in East Asia.

### Citations in the literature

Citations in the literature in the global and East Asia are shown in Figure 2A. It can be seen that the worldwide and East Asian citations overlap highly with their literature growth trends, and also that the East Asian literature citations are growing faster than the worldwide. This indicates that the research literature in East Asia is receiving increasing attention.

Tables 6 shows counts of the top 10 most frequently cited international and East Asian articles.

**Table 6:** Most cited articles on missing persons globally and in East Asia

Scope	Rank	First author	Title	Journal	Year	Number of citations	Citations per year
Global							
	1	Ching T	Opportunities and obstacles for deep learning in biology and medicine	*Journal of Royal Society Interface*	2018	707	176.75
	2	AIQahtani SJ	Brief Communication: the London Atlas of Human Tooth Development and Eruption	*American Journal of Physical Anthropology*	2010	609	50.75
	3	Weissensteiner H	HaploGrep 2: mitochondrial haplogroup classification in the era of high-throughput sequencing	*Nucleic Acids Research*	2016	394	65.67
	4	Carter DO	Cadaver decomposition in terrestrial ecosystems	*Science of Nature*	2007	358	23.39
	5	Cunha E	The problem of aging human remains and living individuals: a review	*Forensic Science International*	2009	349	26.85
	6	Jobling MA	Encoded evidence: DNA in forensic analysis	*Nature Reviews Genetics*	2004	346	19.22
	7	Campobasso CP	Factors affecting decomposition and Diptera colonization	*Forensic Science International*	2001	337	16.05
	8	Mac Donald CL	Diffusion tensor imaging reliably detects experimental traumatic axonal injury and indicates approximate time of injury	*Journal of Neuroscience*	2007	325	21.67
	9	Ritz-Timme S	Age estimation: the state of the art in relation to the specific demands of forensic practice	*International journal of Legal Medicine*	2000	313	14.23
	10	Megyesi MS	Using accumulated degree-days to estimate the postmortem interval from decomposed human remains	*International journal of Legal Medicine*	2005	312	18.35
East Asia							
	1	Salas A	A critical reassessment of the role of mitochondria in tumorigenesis	*PLOS Medicine*	2005	172	10.12
	2	Kidd KK	Developing a SNP panel for forensic identification of individuals	*Forensic Science International*	2006	148	10.50
	3	Sukontasonc K	Forensic entomology cases in Thailand: a review of cases 2000 to 2006	*Parasitology Research*	2007	132	8.8
	4	Walsh, S	Developmental validation of the HIrisPlex system: DNA-based eye and hair colour prediction for forensic and anthropological usage	*Forensic Science International: Genetics*	2014	93	11.63
	5	Wang Q	Stability of endogenous reference genes in postmortem human brains for normalization of quantitative real-time PCR data: comprehensive evaluation using geNorm, NormFinder, and BestKeeper	*International Journal of Legal Medicine*	2012	86	8.60
	6	Inoue S	Rapid simultaneous determination for organophosphorus pesticides in human serum by LC–MS	*Journal of Pharmaceutical and Analysis*	2007	73	4.87
	7	Wang, Z	Screening and confirmation of microRNA markers for forensic body fluid identification	*Forensic Science International: Genetics*	2013	72	8.00
	8	Alkass K	Age estimation in forensic sciences: application of combined aspartic acid racemization and radiocarbon analysis	*Molecular & Cellular Proteomics*	2010	62	4.77
	9	Zubakov D	Human age estimation from blood using mRNA, DNA methylation, DNA rearrangement, and telomere length	*Forensic Science International: Genetics*	2016	57	9.50
	10	Imaizumi K	A new database of mitochondrial DNA hypervariable regions I and II sequences from 162 Japanese individuals	*International Journal of Legal Medicine*	2002	55	2.75

Globally, four of the top 10 cited articles were published in forensic science journals, two were from *Forensic Science International*, another two were published in the *International Journal of Legal Medicine*. All four articles focused on the identification of human remains. The most cited article among the top 10 articles in the WOSCC were “*Opportunities and obstacles for deep learning in biology and medicine*”. Five of the 10 articles were in the review category and focused on deep learning in biology and medicine [36], animal necropsy [37], decomposition of human remains [14], DNA identification of human remains [38], and age estimation from human remains [18]. Taken together, these 10 articles were oriented towards the identification of skeletal remains. The median date of publication of the 10 articles was 2007, with the most recent highly cited literature (one article) published in 2018. This finding could indicate that no new research hotspots focusing on topics such as missing persons have emerged in recent years and that a bottleneck has arisen relating to the exploration and discussion of this topic. In addition, half of the frequently cited literature was in the review category, which also reflects the low number of groundbreaking studies. Moreover, all 10 articles were authored by researchers based in institutions in Europe and the USA, reflecting the currently low levels of discussion on pertinent research in East Asia.

The literature in East Asia as a whole is nevertheless relevant for the identification of human remains. The top 10 cited articles focused on topics that pertain to the identification of skeletal remains. These topics include the exploration of identification techniques, such as the development of single nucleotide polymorphism (SNP) systems for forensic identification [39] and the development of the HIrisPlex system [40]. They also include identification tools, such as microRNA markers for identification [41] and age estimation using mRNA, DNA methylation, DNA rearrangement, and telomere length [42]. Meanwhile, some of the more recent studies from East Asia have also applied novel techniques, such as deep learning and 3D scanning techniques for sex estimation [43]. Such studies indicate that a focus on the application of new technologies relating to the topic of missing persons is beginning to emerge in East Asia, giving new impetus to this type of research in the region.

In addition, of the 3 381 articles in the WOSCC, the four highly cited articles mentioned above were not categorized in the Essential Science Indicators (ESI) field of legal medicine. According to the rules for identifying highly cited articles in the WOSCC, all papers published in the same ESI field, in the same year, were ranked on the basis of the number of citations in descending order from the highest to the lowest, with the top 1% of papers deemed highly cited papers in the WOSCC. Therefore, we speculated that this topic may not currently be a hot topic within legal medicine. Moreover, the issues discussed in these four papers continue to focus on biometrics.

## Discussion

### Summary analysis of relevant publications from East Asia

This bibliometric analysis has revealed some differences between the global and East Asian literatures on missing persons and related topics. The analysis of studies conducted in East Asia showed that although the East Asian studies exhibited some degree of similarity, with many of them focusing on the identification of human remains, there are numerous ethnic groups in East Asia. Consequently, data on identified human remains encompass various ethnicities, such as Manchu, Mongolian, Tibetan, Han, Dong, and Yi [33, 35, 44], and there are even studies that have identified Chinese surnames through short tandem repeat Y-STR profiles of Chinese surnames [45]. There are also some forensic studies have examined domestic animals and insects in East Asia [46, 47]. In addition, given the demographic, geographic, and climatic factors in East Asia, large-scale disasters and MPS feature commonly in the literature on East Asia [20–25], which has explored how human remains can be identified in contexts of large-scale disasters, such as floods and earthquakes. In contrast, only one survey on the identification of human remains following large-scale warfare in East Asia [48] was found for the current study, although some studies are available worldwide, for example, on the wars that took place in Vietnam and Croatia [49, 50]. At the same time, only two social science studies were found among the 238 identified papers focusing on East Asia: (i) an investigation of people who disappeared because of armed conflict [8] and (ii) provisions for sanctioning offenders with mental disorders in China [51].

The exploration of topics such as missing persons in East Asia is geographically specific, but the general direction of the research is in line with the global trend and provides some interesting research material. However, some problems remain. Firstly, the small scale of research in East Asia along with its lack of depth, innovation, and richness has contributed to the low level of discussion of East Asian literature worldwide. The East Asian region has a strong political and cultural identity and a long history, but there are relatively limited existing and publicly available documents that address the issue of missing persons. However, it is encouraging to see that more and more governments and instruction are collaborating on missing persons in East Asia, for example, the remains of Chinese People’s Volunteers martyr. We also found it interesting that the International Committee of the Red Cross has promoted books on related topics worldwide and also in East Asia, such as *Forensic Identification of Human Remains* (https://www.icrc.org/en/publication/4154-forensic-identification-human-remains); such expert advice or workbooks from international organizations can enhance communication and interoperability in the East Asian region and internationally. Secondly, because of language barriers between countries in this region, it is possible to move from a top-level structure to specific issues to meet individualized and social needs and enrich materials on forensic humanitarianism in East Asia. It is also possible to focus on the legacy of historical war events in East Asia, such as the Nanjing Massacre and other historical war events entailing mass casualties to obtain more material rooted in the East Asian context. This analysis indicates that there is scope for the East Asian region to continue to develop research areas with East Asian characteristics, such as MPS, and to explore some interesting topics relating to the social sciences as well as produce more cutting-edge, unique, and innovative research.

### Analysis of the lack of social science research

Although few new hot topics were identified, there is a growing body of studies that provide inputs for identifying human remains, whether from microscopic DNA, RNA, mitochondria, and amino acids or from macroscopic bones and insects. Some studies have begun to explore the use of artificial intelligence to assist in the identification process [52, 53]. Research inputs in all of these areas can facilitate various identification-related tasks. However, it is important to be aware of the strong social connections of each missing or unidentified person that are an important part of their lives and to treat those connections with consideration as a way of respecting their human rights. Over time, their psychological needs will change, and the probability of receiving external support will diminish. The past cannot be admonished, but the future can be traced, and the focus, worldwide, should be on achieving a deeper understanding of the needs of the people concerned. Social science research is challenging, entailing complex statistics, analyses, and summaries, with fewer studies existing within the social science literature compared with the natural sciences literature. Therefore, this analysis only included a few studies from the social sciences.

Some of the more recent studies have provided important inputs in areas such as the potential trauma of collecting the victims’ DNA for their families [54], ethical inquiries [5, 55], and psychological assessments [56, 57]. An increasing body of social science research is currently available and more researchers are starting to focus on this area of research. Strengthening jurisprudential, psychological, and sociological research in this field and presenting more reliable studies will deepen explorations in this field. Such studies can also be combined with social events that are hot topics of discussion, such as the brutalization of children in Canada’s aboriginal residential schools, the crash of a China Eastern Airlines passenger plane on 21 March 2022, and the earthquake and tsunami that occurred in Japan on 11 March 2011. The large-scale human remains left by these events can yield information on the victims relating to their race, sex, and age, which would aid future research on the identification of human remains. In addition, an exploration of the social effects associated with these events is warranted. Some recent studies conducted on the COVID-19 pandemic have focused on this topic [58, 59], applying diverse research perspectives, and discussions have incorporated both the natural and social sciences.

## Conclusions

As an attempt to find missing persons or forensic humanitarian literature, this paper may still lack a list of relevant events in East Asia, but we must recognize the importance of the subject of missing persons. Human beings are an important part of the world; with the progress of society, it is often necessary to review the past and explore human remains, which is an inherent humanitarian task. This bibliometric analysis shows that (i) the research on the issue of missing persons in East Asia has great potential, but lacks the breadth and depth; (ii) the world lacks social science research on the issue of missing persons. In order to address the issues addressed in (i), we need to strengthen cooperation both among East Asian countries and between East Asia and the other parts of the world, and also the East Asian region should continue to preserve its characteristics, effectively addressing current issues; and for (ii), we should continuously promote the development of social science research on missing persons in jurisprudence, ethics, psychology, humanities, etc. and enable more people to participate in social science-oriented discussions focusing on the topic of missing persons. These will make the research on missing persons begin to flourish.
